# COVID-19 Infection Diagnosis: Potential Impact of Isothermal Amplification Technology to Reduce Community Transmission of SARS-CoV-2

**DOI:** 10.3390/diagnostics10060399

**Published:** 2020-06-11

**Authors:** Ameh S. James, John I. Alwneh

**Affiliations:** Good Clinical Practice Research Group, School of Veterinary Science, The University of Queensland, Gatton Campus, Gatton, QLD 4343, Australia; j.alawneh@uq.edu.au

**Keywords:** point of care testing, novel coronavirus, COVID-19, rapid testing, LAMP, RPA, NEAR

## Abstract

The current coronavirus disease 2019 (COVID-19) pandemic is largely driven by community transmission, after 2019 novel Coronavirus (2019-nCoV or SARS-CoV-2) crosses the borders. To stop the spread, rapid testing is required at community clinics and hospitals. These rapid tests should be comparable with the standard PCR technology. Isothermal amplification technology provides an excellent alternative that is highly amenable to resource limited settings, where expertise and infrastructure to support PCR are not available. In this review, we provide a brief description of isothermal amplification technology, its potential and the gaps that need to be considered for SARS-CoV-2 detection. Among this emerging technology, loop-mediated amplification (LAMP), recombinase polymerase amplification (RPA) and Nicking enzyme-assisted reaction (NEAR) technologies have been identified as potential platforms that could be implemented at community level, without samples referral to a centralized laboratory and prolonged turnaround time associated with the standard COVID-19 RT-PCR test. LAMP, for example, has recently been shown to be comparable with PCR and could be performed in less than 30 min by non-laboratory staff, without RNA extractions commonly associated with PCR. Interestingly, NEAR (ID NOW™ COVID-19 (Abbott, IL, USA) was able to detect the virus in 5 min. More so, isothermal platforms are cost effective and could easily be scaled up to resource limited settings. Diagnostics developers, scientific community and commercial companies could consider this alternative method to help stop the spread of COVID-19.

## 1. Introduction

On 30 January 2020, the World Health Organization (WHO) declared SARS-CoV-2 as a global public health emergency [1]. COVID-19 originated from Wuhan province in China, has led to bans on travel and public gatherings and has negatively impacted the world economy [2,3]. COVID-19 is characterized with high morbidity and low mortality, therefore representing great threat, particularly to immunocompromised, elderly people and individuals with pre-existing health problems. The data so far suggest that the virus has a case fatality rate of around 1% [4]. It is thus several times more severe than typical seasonal influenza, with a severity falling somewhere between the 1957 (0.6%) and 1918 (2%) influenza pandemics [4], but it appears to be lower than SARS (9.5%) and Middle East respiratory syndrome (34.4%) [5].

In addition. the average person infected with COVID-19 spreads the disease efficiently to two or three others: an exponential rate of increase. There is also strong evidence that the virus can be transmitted by infected individuals that are asymptomatic or experiencing mild symptoms [6]. This in turns complicates containment or disease control measures compared to diseases caused by other coronaviruses like Middle East respiratory syndrome (MERS-CoV) or severe acute respiratory syndrome (SARS-CoV). Thus, far, COVID-19 has caused approximately ten times as many cases as SARS-CoV in a quarter of the time [7]. Unlike MERS-CoV (camel as intermediate carrier) and SARS-CoV (civet cat as intermediate carrier), the source of COVID-19 remains unknown according to WHO [8,9], but recent studies suggested intermediate carriers could be snakes [9] or pangolins [10], and bats are speculated to be the reservoir host [11].

The median incubation period of COVID-19 is four days (range 0–24 days), and the longest incubation period observed is 24 days [12]. Particularly, some COVID-19 infected individuals do not develop obvious clinical symptoms [13,14]. The long incubation period and asymptomatic infections imply a huge risk of community transmission of SARS-CoV-2 [15,16]. Rapid point of care tests is required to detect the virus in suspected cases at community clinics, hospitals and possibly for house–house testing.

**Figure 1:**
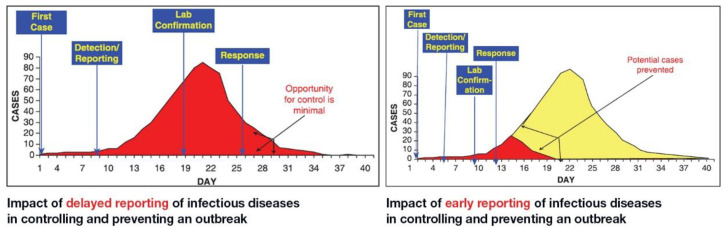
Importance of rapid testing of infectious diseases in controlling an outbreak. The y-axis represents the number of cases, and x-axis indicates the period of detection and response. The red color represents the number of cases without an intervention like testing, and yellow color is the drop in cases due to intervention, drastically reducing the response rate, unlike the graph on the left hand side [22].

Among the measures for stopping the spread of COVID-19 is the availability of reliable and aggressive testing. Presently, samples collected from suspected cases presenting at community clinics and hospitals are being processed at a centralized clinical laboratory, where expensive PCR equipment and technical expertise are available. The turnaround time of the results can be up to 72 h. Such delays in turnaround time could lead to anxiety and the continued spread of the virus. Since self-isolation is not absolutely assured after testing with standard real-time RT-PCR at a centralized clinical laboratory [17]. However, a point of care COVID-19 test can help reduce anxiety, eliminate prolonged turnaround time and reduce the spread of the virus. A point of care device that is rapid, robust, and cost-effective can be used onsite and in the field and does not necessarily require trained personnel to operate [18] is crucial and urgently needed for the detection of SARS-CoV-2. The importance of testing to reduce the spread of an outbreak like COVID-19 has been shown to dramatically control infectious diseases [19,20,21] (Figure 1).

Since the outbreak of COVID-19, regulatory bodies like US Food Drug Administration (FDA) has fast-tracked COVID-19 diagnostics, including isothermal amplification technology platform like Nicking enzyme-assisted reaction (NEAR) as emergency use authorization. This has led to increased uptake of COVID-19 diagnostics from commercial companies, research institutions and in-house development of the tests by clinical laboratories, and these tests are largely based on PCR technology. However, this technology is not completely amenable to onsite or field conditions. In addition, the introduction of rapid immunoassay coupled on lateral flow strip as point of care test has been developed. This test can detect either SARS-CoV-2 antigens or antibodies like IgM and IgG developed against the virus. While this testing method has the advantage of absolutely reducing the turnaround time to less than 10 min and highly cost effective but are likely to be less sensitive and specific based on the experience with influenza viruses rapid immunoassay [23,24,25,26,27].

An alternative and comparable technology to PCR is required to enable a timely detection of SARS-CoV-2. This technology should also be comparable to rapid immunoassay regarding providing point of care testing in less than 30 min and cost effective. This review provides a brief description of common isothermal technologies (loop-mediated amplification and recombinase polymerase amplification) that could be potentially deployed as point of care tests at community clinics and hospitals. These technologies have been proven to have the potential to replace PCR especially in resource limited settings that is characterized with inadequate expertise and infrastructure for PCR setup. There are limited publications on the application of isothermal technologies for testing COVID-19, only few studies have demonstrated its comparability with PCR.

## 2. Isothermal Amplification Technologies (IAT)

IAT is the amplification of nucleic acids at a constant temperature and it does not require thermocycling like PCR. There are different strategies for achieving amplified products (Table 1), and it has been demonstrated in point of care testing as already reviewed extensively [28]. These technologies have been identified as a game changer [28] that will improve the turnaround time to results and ease of use of diagnostic tools for COVID-19, new molecular diagnostic tools using IAT have been developed that can be used as a point of care. Unlike PCR that need to be performed by skilled personnel in a central laboratory, IAT requires little to no training and can be performed at community clinics and hospitals. This means that frontline workers like nurses and medical doctors can perform COVID-19 test when needed at point of care. The IAT technologies offer high sensitivity and specificity when compared with PCR [29,30,31], mainly due to the strand displacement enzymes that are commonly used in these technologies. For example, loop-mediated amplification (LAMP) and NEAR, uses *Bacillus stearothermophilus* DNA polymerase, while DNA polymerase from *Bacillus subtilis* is used by recombinase polymerase amplification (RPA). Their polymerase activity was noted for improved sensitivity and efficiency. Additionally, the use of IAT is less expensive or comparable with PCR-based test. For example, estimates of affordability are less than USD 10 per test and less than USD 500 per piece of equipment [32,33,34].

Several of isothermal technologies have been commercialized and are being used for the diagnosis of human infectious diseases including malaria, tuberculosis, viral hepatitis, chlamydia and gonorrhea [28], using LAMP, RPA, strand displacement amplification and helicase displacement amplification. Among these platforms, loop-mediated amplification is widely applied with more than 1000 peer reviewed publications reported to have utilized LAMP for the detection of human infectious diseases which has been extensively reviewed [33,34,35]. However, the uptake of LAMP as a diagnostic tool for COVID-19 has been very slow, particularly at point of care. Yet, LAMP, RPA and NEAR have recently emerged as potential technologies that could obviate the need for PCR and can be used at community clinics and hospitals in order to identify both symptomatic and asymptomatic individuals, potentially reducing the spread of COVID-19. Excitedly, NEAR was recently approved as emergency use authorization by US FDA for the detection of SARS-CoV-2, and this test is referred to as ID NOW™ COVID-19 (Abbott Laboratories, Illinois, IL, USA).

### 2.1. Loop-Mediated Isothermal Amplification

**Figure 2:**
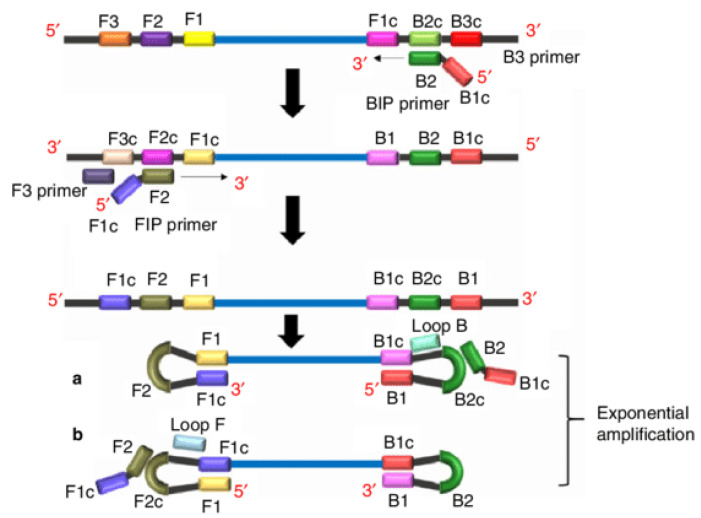
Schematic representation of loop-mediated amplification reaction and its principle. Unlike PCR primer design, LAMP is characterized with four different primers, specifically designed to recognize six distinct regions of the target DNA. Forward inner primer (FIP) consists of a F2 region at the 3’-end and an F1c region at the 5’-end. While the F3 primer (forward outer primer) consists of a F3 region which is complementary to the F3c region of the template sequence. The Backward Inner primer (BIP) is made up of a B2 region at the 3’-end and a B1c region at the 5’-end. B3 primer (backward outer primer) consists of a B3 region which is complementary to the B3c region of the template sequence. In regards to LAMP reaction, amplification begins when F2 region of FIP anneals to F2c region of the target DNA and initiates complementary strand synthesis, and F3 primer anneals to the F3c region of the target and extends, displacing the FIP linked complementary strand. This displaced strand forms a loop at the 5’-end, which provides the template for BIP, and B2 anneals to B2c region of the template. DNA synthesis is initiated, which results in the formation of a complementary strand and opening of the 5’-end loop. Subsequently, B3 anneals to B3c region of the target DNA and extends, displacing the BIP linked complementary strand, which forms a dumbbell-shaped DNA. The nucleotides are added to the 3’-end of F1 by Bst DNA polymerase, which extends and opens up the loop at the 5’-end. The dumbbell-shaped DNA is converted to a stem–loop structure (a and b), which initiates LAMP cycling (second stage of LAMP reaction). The amplicons formed are a mixture of stem–loop and cauliflower-like structures with multiple loops [49].

The LAMP reaction mechanism simply involves nucleic acids unwinding, amplification and elongation. This approach has been described in an animation by Eiken Chemical (http://loopamp.eiken.co.jp/e/lamp/anim.html), which is really helpful in understanding how the principle works. Essentially, LAMP uses a Bst DNA polymerase with strand-displacement activity, coupled with two inner primers (FIP, BIP) and outer primers (F3, B3) that recognizes six separate regions on a DNA template. Additional loop primers (LF, LB) may be added to speed up the reaction by binding to and amplifying newly formed loop amplicons in the reaction. These additional primers further enhance the sensitivity and specificity of the reaction (Figure 2). LAMP reactions are incubated at a constant temperature ranging from 60–65 °C with time to amplification (that is, a positive LAMP result) in less than 60 min [39]. LAMP and NEAR are also compatible for the detection/amplification of RNA templates directly with Bst 3 DNA polymerase (New England Biolabs, Melbourne, Australia) or with a reverse transcriptase in the reaction [48].

**Figure 3:**
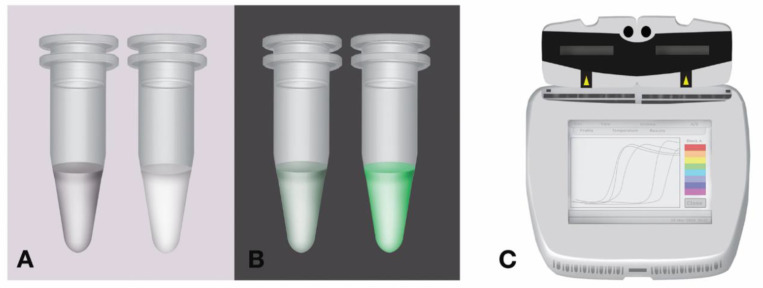
Schematic naked-eye visualization strategy of LAMP reaction. (**A**) Detection of LAMP reaction by turbidity. Left tube, without turbidity (negative); right tube, with turbidity (positive); (**B**) detection of LAMP reaction by fluorescence using calcein. Left tube, without green color (negative); right tube, with green color (positive); (**C**) real-time detection of LAMP reaction using a portable equipment like Genie^®^ II. The equipment is portable and robust for point of care testing and it uses 24-h rechargeable battery. This equipment is available from OptiGene Limited, Horsham, UK.

A LAMP positive reaction can be determined visually and quantified based on turbidity, colorimetry and fluorimetry. The insoluble byproduct, magnesium pyrophosphate, which is formed during the LAMP reaction, can be seen with the naked eye (cloudiness) (Figure 3A) [34]. Recently, this endpoint detection was improved by using calcein, a fluorescein complex, which fluoresces (visible color change) in the presence of magnesium pyrophosphate (Figure 3B) [34,50]. Nucleic acid intercalating dyes including SYBR Green can be added to detect (visible color change) and quantify the loop amplicons formed. This detection system allows real-time monitoring of the reaction using isothermal equipment equipped with fluorescence detectors. This commercial equipment has been optimized and is highly adaptable across a wide range of IAT including NEAR, LAMP and RPA (Figure 3C).

There is paucity in the literature on the application of IAT for COVID-19 diagnosis. The studies so far showed that one-step RT-LAMP test is comparable with RT-qPCR. Lin Yu et al. [29] showed RT-LAMP test could detect synthesized RNA equivalent to 10 copies of SARS-CoV-2 virus, and the clinical sensitivity was 97.6% (42/43) with samples validated by RT-qPCR. Their test targeted *ORF1ab* region of the virus and it could be performed between 20–40 min. While Renfei Lu et al. [30], targeted *RdRp* gene and showed limit of detection as 3 copies of synthesized SARS-CoV RNA and 100% sensitivity (17/17) as determined by RT-qPCR in 40 min. In addition. their test did not cross react with 15 clinical samples that were positive for respiratory viruses like enterovirus, respiratory syncytial virus A and B groups, parainfluenza viruses type 1–3, influenza A–C, human rhinovirus, human metapneumovirus, adenovirus, bocavirus and human coronavirus strains. Unlike other studies, Laura Lamb et al. [31], detected 1.02 fg of SARS-CoV-2 fragments and used simulated patient samples by spiking saliva, urine, serum, oropharyngeal and nasopharyngeal swabs with fragments of synthetic SARS-CoV-2 without extractions. Their reaction test time was less than 30 min and it was highly specific as it does not cross react with MERS, beta-coronavirus England-1 or Murine hepatitis virus when spiked into patient samples.

### 2.2. Recombinase Polymerase Amplification

**Figure 4:**
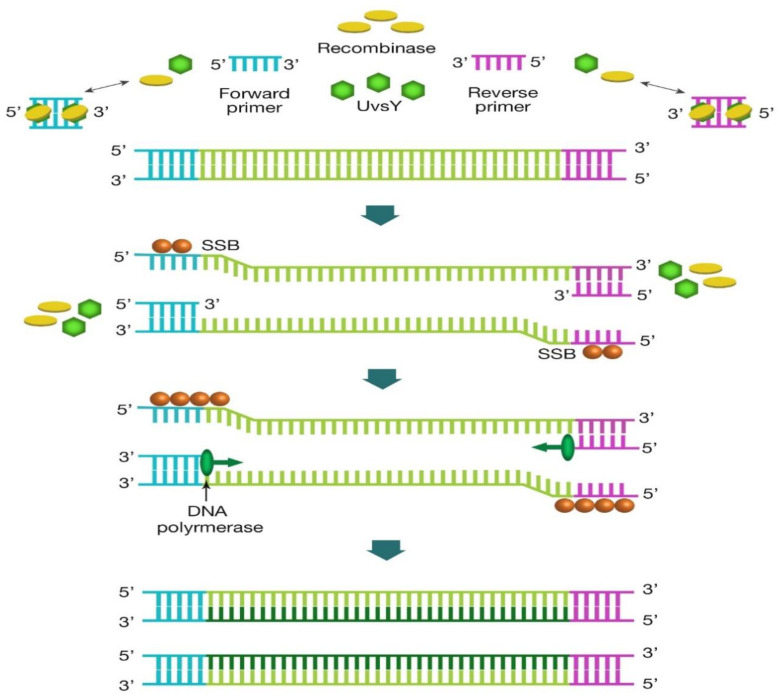
Recombinase polymerase amplification schematic representation. It begins with the binding of recombinase (T4 uvsY and uvsX; green diamonds and orange circles, respectively) to forward and reverse primers, which forms a complex that search for homologous sequences in double stranded DNA. Strand exchange reaction occurs once the homology is found. The single strand binding proteins (SSB, T4 gp32 protein; brown circles) aligns to unwound DNA strand, allowing DNA polymerase (*Bacillus subtilis* Pol I, Bsu; green circles) to initiate template amplification using the two primers, forming two double stranded DNA. The repetition of the cycle leads to exponential amplification.

**Figure 5:**
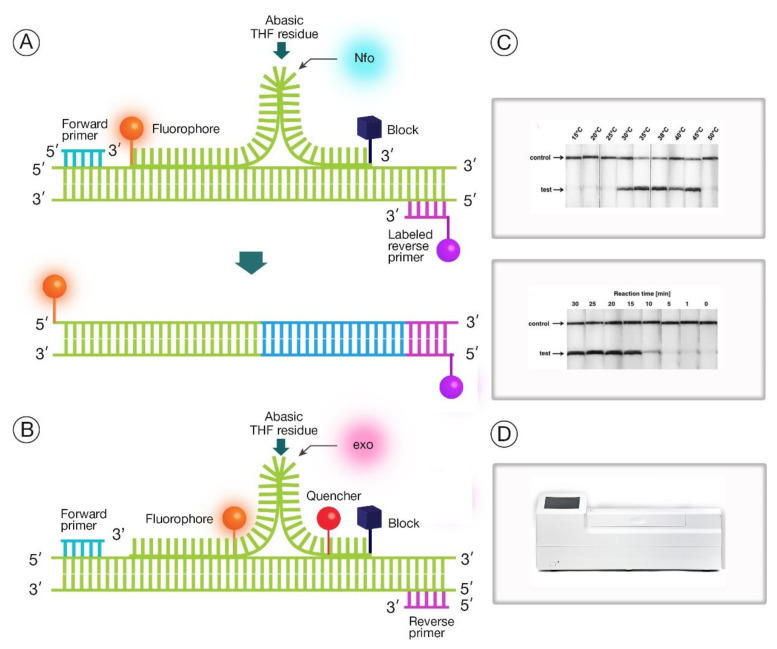
RPA detection mechanism. (**A**) The TwistAmp nfo is for lateral flow detection strategy, while (**B**) exo probe is for real-time detection. The probe annealed to double stranded DNA has a 3′ block (dark blue) that prevents extension. The *Escherichia coli* endonuclease IV (nfo) or exonuclease III (exo) recognizes and cleaves the tetrahydrofuran (THF) residue (as indicated with the arrow) within the probe, detaching the 3′-end block. This process helps the integration into the amplified products through Bsu polymerase elongation from the 3′-end hydroxide; (**A**) Regarding nfo amplification, fluorophore labeled amplicons (for example, with fluorescein amidites and biotin dyes) can be detected visually using lateral flow strips. This sandwich format allows the fluorophore (bright orange) to be captured through anti-fluorophore conjugated gold nanoparticles. It also can detect a second label like biotin (purple) by binding to a streptavidin detection line; (**B**) Regarding exo amplification, fluorescent signals are generated when exonuclease III (exo, pink) cuts the THF site like the nfo, separating the fluorophore (bright orange) from the quencher (red); (**C**) The lateral flow coupled with RPA nfo reaction can be performed within a broad range of temperatures (top) and a positive test is observed visibly after 10 min (bottom) [54]; (**D**) The exo fluorescent signals are detected by a real-time device, such as the T16-ISO equipment from TwistDx, Cambridge, UK.

The amplification of nucleic acids using RPA is faster than LAMP, at 37 °C or less [51]. RPA employs recombinase proteins that forms a complex with primers that scan for homologous sequences and unwind double stranded template (Figure 4) [28,43]. The amplified products can either be monitored in real time or sandwiched on a lateral flow strip, and the two commonly used detection systems are possible with unique probes which are the same in design except for internal modifications (Figure 5) [28]. Usually, the test design by the diagnostic developer determines the choice of probe; the exo probe is used for real-time monitoring, while nfo is suitable for lateral flow strip. The recombinase proteins and monitoring devices (including lateral flow strips) are commercially available from companies like TwistDx, Cambridge, UK. Hence, the developer only needs to design and screen primers and probe that targets pathogen of choice. Interestingly, PCR primers could also be adopted for RPA assay development, unlike LAMP primers. To date, no study has been demonstrated using this technology for COVID-19 diagnosis, but RPA has been deployed during the last Ebola virus outbreak that was declared as a global public health epidemic by WHO [52].

### 2.3. Nicking Enzyme-Assisted Reaction

**Figure 6:**
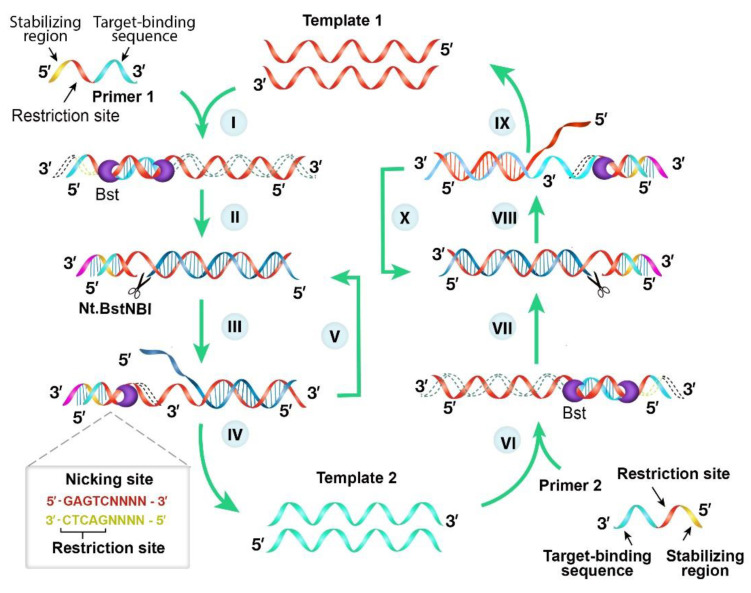
Schematic representation of nicking enzyme-assisted reaction. Unlike PCR primers, NEAR primers are uniquely designed for a successful reaction. Each primer has three regions: restriction site (5′-GAGTCNNNN-3′, for example, Nt. BstNBI), stabilizing region and a target-binding region that is complementary to the target nucleic acid strand. First, the forward primer (primer 1) anneals with template 1 at the target-binding region and extended from its 3′ end by DNA polymerase. This results in the formation of an intermediate strand, complementary to template 1 (step I). At the same time, the nicking endonuclease recognizes the asymmetric restriction site (5′-GAGTC-3′) cleaves the strand with four base pairs after the recognized sequence introduces a new nick site (step II). At the nick site, DNA polymerase initiates another extension and displace the intermediate strand generated in step I (step III and IV). This displaced strand (template 2) carries the target-binding sequence complementary to reverse primer (primer 2), and DNA polymerase extends from the 3′ end of this primer (step VI). Again, nicking endonuclease recognizes and cleaves the restriction site, and the polymerase enzyme extends and displaces the initial template strand (template 1) from the nick site, which is recovered (step VII and IX) for another cycle, starting from step I. The target template is exponentially amplified by repeating this cycle of events [53].

NEAR is driven by two enzymes (nicking endonuclease and DNA polymerase) and with reaction buffer, deoxyribonucleotide triphosphate and primers, a linear amplification of DNA template is achieved. This amplification eventually leads to exponential increase of amplified products [53], and it can be coupled on a fluorometer. Briefly, NEAR reaction occurs at 60 °C and it involves five steps: (a) the DNA template is hybridized with primers that are conjugated with nicking endonucleases restriction site, cleaving the double stranded DNA (b) from the 3′ end of the primer, DNA polymerase extends nucleotides forming a double stranded DNA; (c) nicking endonucleases identifies the restriction site on the primers and nicked one of the strands, exposing the 3′end; (d) DNA polymerase extends from the nicked site by employing uncleaved strand as a template for a new double stranded DNA, displacing the former strand for another cycle of DNA synthesis, but restriction site is recovered on the newly synthesized double stranded DNA; (e) These steps continuously amplified targeted DNA template via cleavage, extension and recovery (Figure 6).

## 3. Future Direction of IAT for COVID-19 Diagnosis

The potential of a new diagnostic technology is usually demonstrated when matched with an existing and well-established technology. For a COVID-19 IAT to be considered acceptable for diagnostic purposes at community clinics and hospitals, research must not only demonstrate its diagnostic sensitivity and specificity, which is the common practice, but should also consider factors that make up an ideal diagnostic test in such a setting. There are criteria that guide implementation of such a rapid molecular test: a rapid clinical decision, test safety and usage by medical doctors, nurses and non-laboratory staff, quick result during a patient visit and cost effectiveness when compared with PCR technology. IAT like LAMP, NEAR and RPA have the potential to fulfil majority of these criteria, but more studies are urgently needed in order to validate these technologies for COVID-19 diagnosis. In spite, the advantages of IAT, the lack of internal inhibition control could result to duplication of reactions during testing. In addition, the complexity of primer design of LAMP test, for example, can prove difficult in target site selection and resolution [55].

### 3.1. Safety Measures for Sample Process and IAT Testing

Normally, respiratory samples collected as either nasopharyngeal or oropharyngeal swabs in viral transport medium are processed in class II or III biologic safety cabinet [56]. This level of containment is not feasible at community clinics and hospital. Collecting the swab samples directly into lysis buffer containing inactivating agent like guanidinium and non-denaturing detergent would be suitable for direct testing with IAT like LAMP. The guanidinium and detergents are able to inactivate any viable coronavirus and prevent RNA degradation, respectively [57,58,59], combined with personal protective equipment (latex hand gloves, laboratory coat, appropriate face mask and eye goggle) makes SARS-CoV-2 IAT testing safe for non-laboratory personnel at clinics and hospitals.

LAMP has already been shown to possess the ability to withstand inhibitory substances present in cell-based clinical samples, and it was associated to its complex primer assembly on the DNA or its complementary strand [39]. Unlike LAMP, RPA is yet to demonstrate direct detection of target from clinical samples, this means that RPA could probably be inhibited with cell-based samples. Respiratory samples for COVID-19 are free of cells suggesting RPA potential to directly test this type of sample. For example, RPA has been demonstrated to directly test urine samples for *Chlamydia trachomatis* [60]. However, heating of the respiratory samples should be avoided to prevent RNA degradation which could impact on the outcome of the IAT test outcome.

### 3.2. Development of Multiplex Detection of SARS-CoV-2

Presently, at least two different targets of SARS-CoV-2 are required for confirmation of COVID-19. This is to avoid cross reaction with other potential endemic coronaviruses and genetic drift of SARS-CoV-2 [56]. These targets could be envelope (*E*), helicase (*Hel*), transmembrane (*M*), structural proteins (envelope glycoproteins spike (*S*) and nucleocapsid (*N*) [61,62,63]. Or, species-specific accessory genes that are required for viral replication (RNA-dependent RNA polymerase (*RdRp*), hemagglutinin-esterase (*HE*) and open reading frames *ORF1a* and *ORF1b* [14,17,61,62,64]. Studies on SARS-CoV-2 are still evolving and the extent of the virus mutations is still not clear. Notwithstanding, an ideal nucleic acid test design should include at least one conserved region and one specific region in order to mitigate against the effects of SARS-CoV-2 genetic drift as the virus evolves within new populations [63]. The US Centers for Disease Control recommends two nucleocapsid protein targets (*N1* and *N2*) [64], while WHO recommends first line screening with the *E* gene test followed by a confirmatory test using the *RdRp* gene [17]. For IAT to be cost-effective and efficient, a multiplex test targeting two genes will be appropriate for clinical decision. To date, the published RT-LAMP tests did not evaluate SARS-CoV-2 using two target genes. However, these RT-LAMP studies and commercialization of IAT are urgently required to help prevent the spread of the virus at community level. There is high expectation from diagnostic developers that IAT hold promise to reduce the spread of COVID-19 at resource limited settings, where central laboratories are far away from the transmission of the disease.

LAMP, NEAR and RPA do have potentials for their simplicity, sensitivity, specificity and quick result, as demonstrated by incorporation with other technologies like lateral flow strip and portable detection reader. These IAT shows their ability to be easily amenable to different detection platforms. Notwithstanding, clinical evaluation are expected for multiplexing of the targeted genes in order to further assess the capability of IAT to confirm the diagnosis of COVID-19. Like PCR, only a pair of primer is required for RPA reaction, making the assay design relatively easy; however, the multiplexing ability is yet to be fully explored. Notwithstanding, RPA has been used to multiplex food pathogens like *Staphylococcus aureus*, *Vibrio parahaemolyticus* and *Salmonella enteritidis* coupled on a lateral flow strip, and the test line intensities were quantified using a test strip reader [65]. It will be useful to see this recent strategy deployed for SARS-CoV-2 multiplexing as well.

Currently, there is urgent demand for tests that could confirmed cases of COVID-19 and not just for screening, so it will be helpful to know the extent of contribution of LAMP, NEAR and RPA in this regard. However, LAMP has been used to multiplexed influenza A and B [66] with Genie II fluorometer as the detection platform (Figure 3C). This device shows amplification signals and time to completion of reaction in real time. Sample melting temperature (Tm) is also shown on the device. Mahony James et al. [66] employed the Tm strategy in the differentiation of influenza A and B, and it was easily differentiated because the Tm did not overlap. This strategy could be deployed for SARS-CoV-2 multiplexing as well, but it will require a technical training for point of care testers without a background in nucleic acid testing.

### 3.3. Integrating Sample Preparation, Amplification and Detection to Reduce Cross Contamination

The advantage of real-time RT-PCR testing is the amplification and analysis are done simultaneously in a closed system to reduce false-positive results commonly linked with amplicons, and the capacity of the test to process up to 96 samples at once. Among all the nucleic acids tests, the integration of sample preparation, amplification and detection into a single platform has been a challenge, especially with sample preparation [67]. As a result, sample preparation is still processed in isolation of amplification and detection by most test applications. Interestingly, RT-LAMP could directly be performed on clinical samples without RNA extractions, and this has been demonstrated with the published COVID-19 RT-LAMP studies [29,30,31], but the throughput is 16 samples if Gene II fluorometer is used for amplification and detection, which could slow the testing process considering the high demand for COVID-19 test at point of care.

In addition, the inclusion of a thermolabile uracil-DNA glycosylase (UNG) in LAMP reactions could help prevent cross-contamination between samples, as already demonstrated by Laura Lamb et al. [31]. This approach simply works by introducing uridine triphosphate (UTP) in the reaction along with thymidine triphosphate (TTP) and the other nucleoside triphosphates. During amplification, few UTP replaces TTP creating a mix of UTP and TTP amplicons. The UTP amplified products are cleaved by UNG to prevent it from being re-amplified in the next cycle. The introduction of UNG in isothermal reactions especially when coupled with lateral flow strips, makes the system suitable for point of care, where non-laboratory staff may not be proficient enough to handle nucleic acid testing.

As the current COVID-19 pandemic continues, urgent point of care tests like the ID NOW™ COVID-19 (Abbott Laboratories, Illinois, IL, USA), and comparable with the standard RT-PCR are needed. LAMP, NEAR and RPA have the potentials to reduce the spread of SARS-CoV-2, but diagnostic developers, scientific community and commercial companies are required to make contributions in this regard. Importantly, our review is limited because it is a rapidly developing area in regards to COVID-19 diagnosis, and the presented information is at the time of publication, and IAT for SARS-CoV-2 testing may keep evolving until the pandemic is over.

## Figures and Tables

**Table 1:** Initial publications of isothermal amplification technologies strategies for nucleic acid amplification.

Isothermal Amplification Technology	Method for Denaturing Nucleic Acids	Estimated Reaction Time	Reference
Nucleic acid sequence-based amplification	Thermal	90 min	[36]
Transcription-mediated amplification	Thermal	1–2 h	[37]
Multiple displacement amplification	Enzymatic	8–10 h	[38]
Loop-mediated amplification	Enzymatic	<1 h	[39]
Helicase-dependent amplification	Enzymatic	2 h	[40]
Rolling circle amplification	Enzymatic	1 h	[41]
Signal-mediated amplification	Thermal	2 h	[42]
Recombinase polymerase amplification	Enzymatic	10 min	[43]
Self-sustained sequence replication	Thermal	<1 h	[44]
Ramification amplification	Enzymatic	1 h	[45]
Strand displacement amplification	Thermal	2 h	[46]
Nicking enzyme-assisted reaction	Enzymatic	15–30 min	[47]
